# Mesenchymal stem cells rescue cardiomyoblasts from cell death in an in vitro ischemia model via direct cell-to-cell connections

**DOI:** 10.1186/1471-2121-11-29

**Published:** 2010-04-20

**Authors:** Attila Cselenyák, Eszter Pankotai, Eszter M Horváth, Levente Kiss, Zsombor Lacza

**Affiliations:** 1 Institute of Human Physiology and Clinical Experimental Research, Semmelweis University, H-1094, Tűzoltó utca 37-47, Budapest, Hungary

## Abstract

**Background:**

Bone marrow derived mesenchymal stem cells (MSCs) are promising candidates for cell based therapies in myocardial infarction. However, the exact underlying cellular mechanisms are still not fully understood. Our aim was to explore the possible role of direct cell-to-cell interaction between ischemic H9c2 cardiomyoblasts and normal MSCs. Using an in vitro ischemia model of 150 minutes of oxygen glucose deprivation we investigated cell viability and cell interactions with confocal microscopy and flow cytometry.

**Results:**

Our model revealed that adding normal MSCs to the ischemic cell population significantly decreased the ratio of dead H9c2 cells (H9c2 only: 0.85 ± 0.086 vs. H9c2+MSCs: 0.16 ± 0.035). This effect was dependent on direct cell-to-cell contact since co-cultivation with MSCs cultured in cell inserts did not exert the same beneficial effect (ratio of dead H9c2 cells: 0.90 ± 0.055). Confocal microscopy revealed that cardiomyoblasts and MSCs frequently formed 200-500 nm wide intercellular connections and cell fusion rarely occurred between these cells.

**Conclusion:**

Based on these results we hypothesize that mesenchymal stem cells may reduce the number of dead cardiomyoblasts after ischemic damage via direct cell-to-cell interactions and intercellular tubular connections may play an important role in these processes.

## Background

Cardiovascular diseases represent an enormous medical and social burden [1,2] and the pathophysiology of most of these diseases, such as myocardial infarction or heart failure, involves death of cardiac myocytes leading to a loss of functional tissue. Cell based therapies are commonly believed to be the next generation of therapies for replacing such lost tissue [3-5]. Several in vivo animal and human studies have found that implantation of various cell types, typically bone marrow derived stem cells, into damaged myocardium improved cardiac performance. Also where the experimental protocol allowed, surviving grafted cells were detected in the myocardium [6,7], suggesting that grafting is an effective treatment of acute myocardial infarction [8]. However, the extent of the beneficial effect, the optimal cell type and number, the best method of administration, and the mechanism of action need to be further evaluated [9].

One important issue is the exact mechanism of action, in other words, the interaction between graft and host. Paracrine factors, transdifferentiation and cell fusion are the three generally accepted hypotheses explaining the beneficial effects of stem cell grafting. Paracrine factors through various effects, such as increased angiogenesis or modulation of postinfarct remodeling, may represent an important aspect of the benefits [10,11]. On the other hand, several studies have found that co-culture of cardiomyocytes with pluripotent stem cells resulted in transdifferentiation of these cells into cardiomyocytes, which raised the hope that in vitro cultured tissue blocks can later be used for cardiac repair [12,13]. However, although it is possible to construct a tissue in vitro this does not mean that its building blocks will perform similarly when implanted in vivo [14]. Indeed, recent investigations found difficult to reproduce transdifferentiation and that bone marrow derived cells generate cardiomyocytes not by transdifferentiation but rather through cell fusion [15,16]. Alvarez-Dolado et al demonstrated that bone marrow derived cells fused with cardiomyocytes [17], but the importance of cell fusion events was questioned by an other investigation [18]. Even in studies which found morphologically adequate new cardiomyocytes, the volume of this newly formed tissue seemed to be inadequate to account for the functional benefits. Other hypotheses have also emerged to resolve the apparent controversy among the clinical findings and the cell culture studies, such as the most recently proposed partial cell fusion through direct cell-to-cell interactions. This novel intercellular communication route depends on short cell-to-cell interactions, during which the two connected cells exchange membrane and organelle parts such as mitochondria or other cytoplasmatic components [19]. Recently, it was reported that cardiomyocytes and human mesenchymal stem cells appear to communicate through small diameter nanotubes, and mitochondria can migrate from MSCs to cardiomyocytes [20]. However, the physiological purpose of this constantly changing nanotubular network and its possible role during ischemic conditions is unclear. We hypothesized that stem cells and post-ischemic cardiomyoblasts interact with each other via this novel mechanism and that this mechanism may play a role in the beneficial effect of stem cell transplantation.

The aim of our study was to examine the possibility of rescuing ischemically damaged H9c2 cardiomyoblasts from cell death by adding mesenchymal stem cells to the cultures after ischemia. Furthermore we investigated the importance of direct cell-to-cell interactions during co-cultivation of these cells.

## Methods

### Isolation and culture of cardiomyoblasts and MSCs

H9c2 rat cardiomyoblasts were obtained from ATCC (Wesel, Germany) and expanded in high glucose (4.5 g/L) DMEM containing 10% fetal bovine serum, 4 mM L-glutamine, 100 U/ml penicillin and 100 μg/ml streptomycin. Mouse mesenchymal stem cells (MSCs) were harvested from the femur of C57Bl/6 mice. Isolation and primary culture was performed according to Tropel's method with small alterations [21]. Briefly, animals were anaesthetized with pentobarbital (ip, 50 mg/kg, Nembutal, Ovation, Deerfield, IL, USA), lower limbs were removed and femurs were cleaned of tissue. Bone marrow was collected by flushing femurs with low glucose DMEM containing 10% fetal bovine serum, 2 mM L-glutamine, 100 U/ml penicillin and 100 μg/ml streptomycin. Cells were centrifuged at 1200 rpm and plated in a T75 flask. After 4-5 days, non-adherent cells were removed by washing twice with PBS and adherent cells were then cultured in low glucose DMEM complete medium. Characterization of the cultured MSCs showed that these cells were strongly positive for the specific surface antigen Sca-1 and negative for differentiation markers of other cell lineages (CD34, CD3ε, CD45R/B220, CD11b, 6G, and TER-119) and were able to differentiate into the osteoblast and adipocyte lineages in vitro, verifying the MSC phenotype [22]. Cell culture media was replaced every 2-3 days thereafter. All investigations conformed to the Guide for the Care and Use of Laboratory Animals published by the National Institutes of Health (NIH Publication No. 85-23, Revised 1985), and were approved by the local ethics committee.

### In vitro ischemia model

Ischemia-reperfusion was simulated in vitro by performing oxygen glucose deprivation (OGD) on H9c2 cell cultures. Cells were incubated in glucose-free DMEM in an atmosphere of 0.5% O_2 _and 99.5% N_2 _for 150 minutes. This procedure was performed on the stage of the confocal microscope (PECON incubation system, Erbach-Bach, Germany) allowing the observation of the cells during OGD. To evaluate cell viability we used calcein-AM (excitation/emission 494/517 nm) to identify live cells, and ethidium-homodimer (excitation/emission 528/617 nm) to stain dead or damaged cells [23].

### Co-cultivation of cardiomyoblasts and MSCs after OGD

H9c2 cells and MSCs were labeled before co-cultivation with Vybrant DiO (excitation/emission: 488/501 nm) and DiD (excitation/emission: 633/665 nm) (Molecular Probes, USA) membrane dyes in a dilution of 1:200 according to the manufacturer's description for 30 minutes at 37°C. The DiO-labeled H9c2 cells were plated in 12 well-plates at a density of 30,000 cells/well in 2 ml culture medium. Cells were subjected to 150 min OGD, then the medium was changed and 20,000 DiD-labeled MSCs/well were added to the damaged H9c2 cells 30 minutes after the end of OGD either directly or in cell culture inserts (0.4 μm pore size, Becton Dickinson, NJ, USA). H9c2 cells not receiving MSCs after OGD were used as controls. Cells were cultivated for a further 24 hours, then labeled with the dead cell stain ethidium homodimer (4 μM, 30 minutes, and 37°C), then investigated either with confocal microscopy (Zeiss LSM 510 META, Carl Zeiss, Jena, Germany) or with flow cytometry (BD FACSCalibur™, Becton Dickinson, NJ, USA).

### Confocal microscopy and flow cytometry

Time lapse video microscopy was performed during and after in vitro ischemia to investigate morphological changes and possible interactions among the cells over time (1 picture/3 minutes). The H9c2 cardiomyoblasts and MSCs were co-cultured on 42 mm coverslips and stained with Vybrant DiO and DiD, respectively. In experiments to observe mitochondria, all cells were stained after OGD with MitoTracker Red (Molecular Probes, USA) in a dilution of 1:2000 for 10 minutes at 37°C according to the manufacturer's description. Flow cytometric measurements were performed on single cell suspensions of trypsinized (0.05% trypsin-EDTA) cell cultures 24 hours after OGD and on normal cell cultures (control) using BD FACSCalibur™. DiO-labeled H9c2 cells were identified and gated. Fluorescence data were collected using logarithmic amplification until 10,000 counts were reached.

### Evaluation of fluorescence images and statistical analysis

The evaluation of confocal images for live and dead cells selected by morphology and fluorescence was performed with ImageJ software (National Institutes of Health, USA). In case of co-cultures, MSCs were distinguished from H9c2 cells due to their Vybrant DiD cell labeling. The ratio of dead cells was evaluated in 4 independent fields of view (objective 10×) for each culture in a blind fashion. The evaluation of flow cytometry files was carried out using BD CellQuest™ Pro (Becton Dickinson, NJ, USA). Statistical analysis of data was carried out using one-way analysis of variance with Tukey's multiple comparison post hoc test. Data are expressed as mean ± SEM.

## Results

### In vitro ischemia model

**Figure 1:**
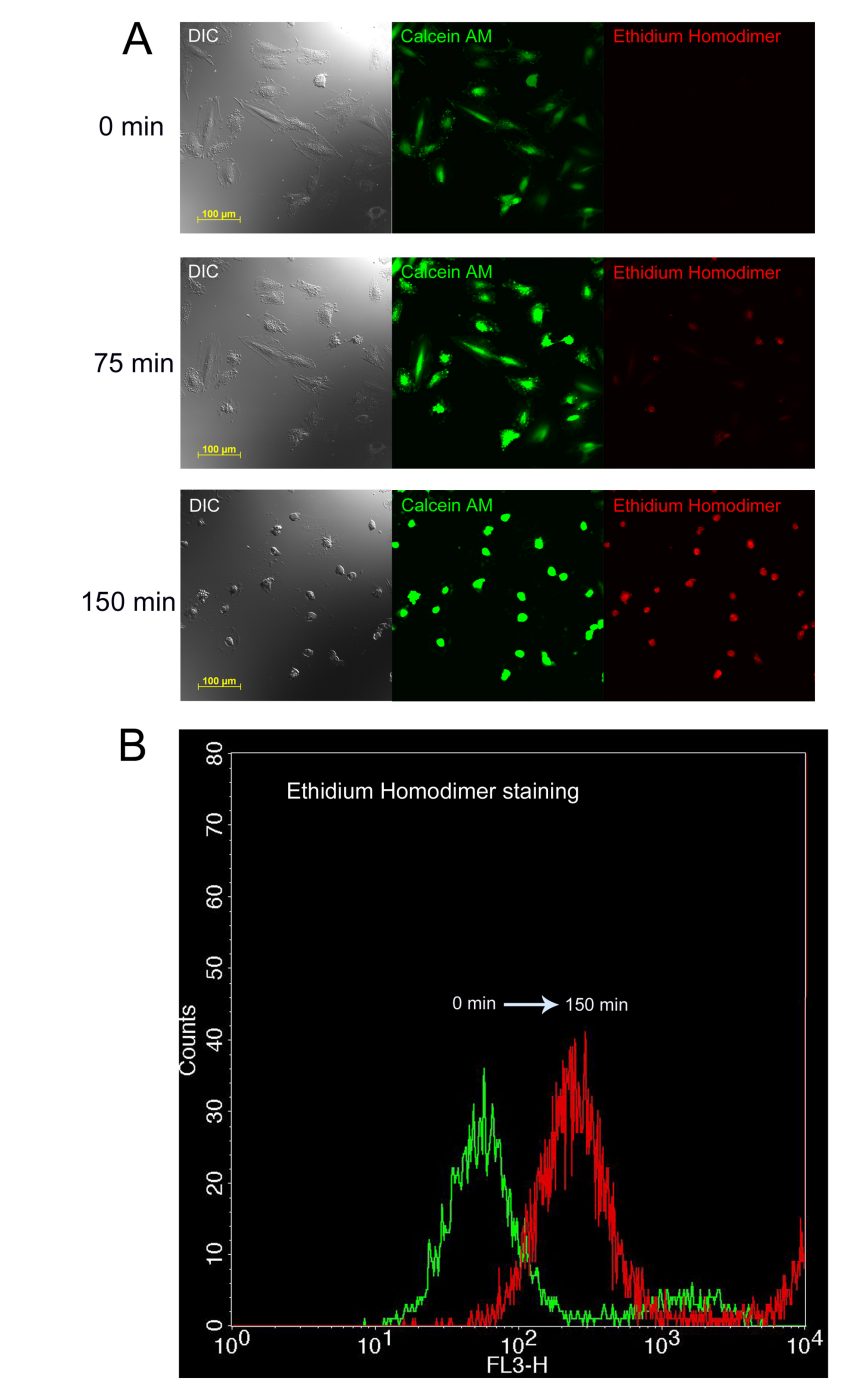
**Ischemia model on cardiomyoblasts**. **(A) **Follow up of OGD on cardiomyoblasts. Cells were stained with calcein-AM (ex/em 494/517 nm) for live cells (green) and ethidium homodimer (ex/em 528/617 nm) for dead cells (red). **(B) **Flow cytometry analysis of control and ischemic cardiomyoblasts labeled with ethidium homodimer after OGD. The green curve represents the control cardiomyoblasts and the red shows the ischemic cardiomyoblasts. The complete rightward shift of the red curve based on these representative data indicates that OGD increased the number of dead cells nearly maximally.

The optimal duration of OGD to induce cell damage was 150 minutes (Figure 1A and additional file 1: video1.mov). This result is based on microscopic observations of morphological changes in the cell shape and on ethidium homodimer staining which determined whether a particular cell was dying. Flow cytometric analysis was also used to determine that the selected time interval was sufficient to injure the majority of the cardiomyoblasts (Figure 1B).

Experiments showed that 4-6 hours was not adequate for the added MSCs to attach to the surface of the 12-well plates and exert their effect, 48 hours produced a culture overgrown by cardiomyoblasts and MSCs (data not shown). Therefore, the time point for microscopic evaluation and flow cytometry analysis subsequent to addition to MSCs was determined to be 24 hours.

### Co-cultivation of cardiomyoblasts and MSCs after OGD

**Figure 2:**
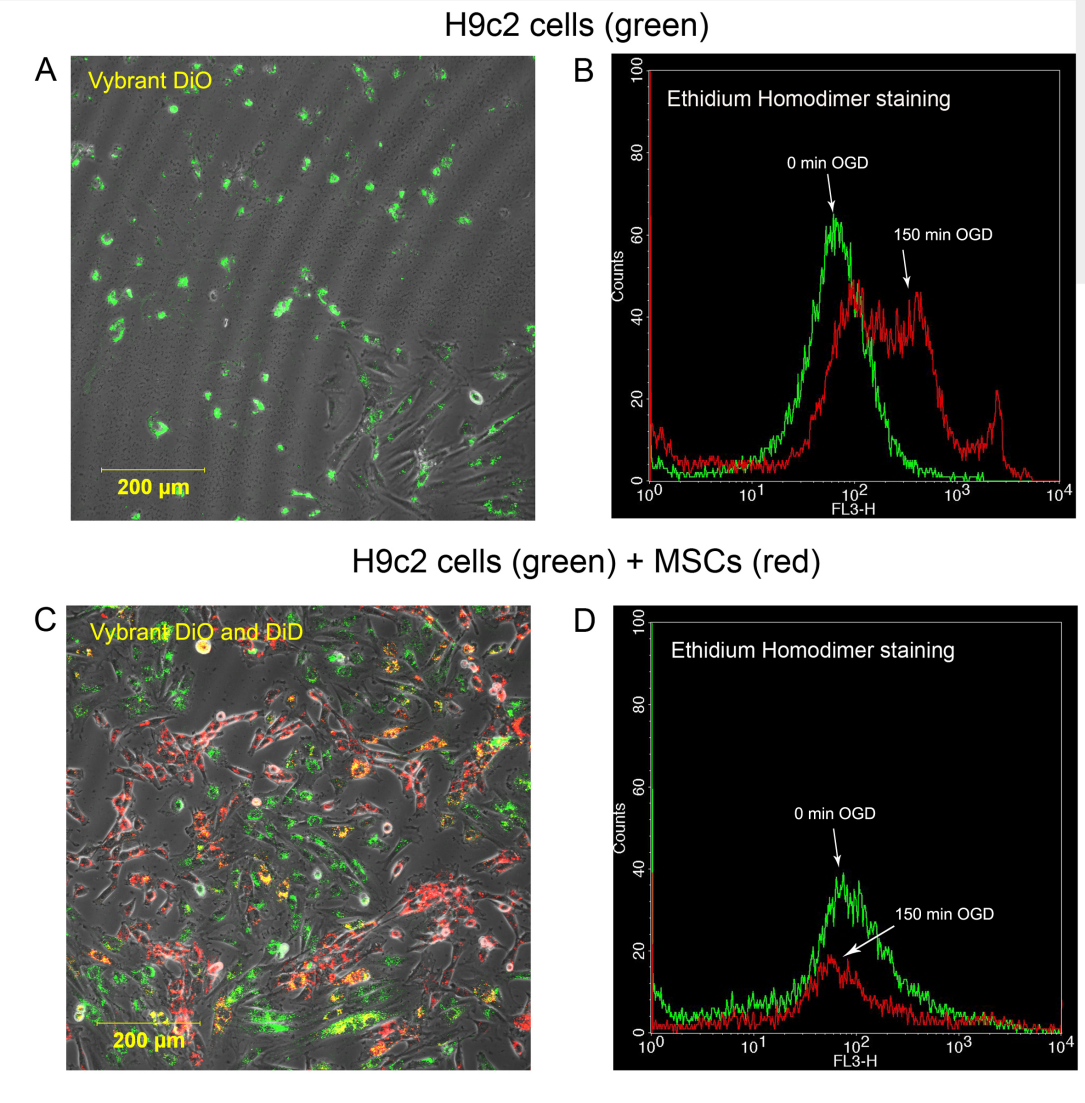
**Morphology and viability of H9c2 cells 24 hours after OGD**. **(A) **DiO-labeled H9c2 cells without MSCs observed 24 hours after OGD were predominantly rounded up and stained with ethidium homodimer indicating cell death in progress. **(B) **Flow cytometry analysis of control and ischemic H9c2 cells cultured for 24 hours after OGD labeled with ethidium homodimer (ex/em 528/617 nm) showed that the number of dead H9c2 cells was elevated compared to the control group (median fluorescence from 19 to 65 units). **(C) **Co-cultivation of DiO-labeled H9c2 (ex/em 488/501 nm) cells and DiD-labeled MSCs (ex/em 633/665 nm) for 24 hours after OGD showed that the morphology of ischemically damaged cells were normal after 24 hours. **(D) **Flow cytometry analysis revealed that after co-cultivation of cells the number of dead H9c2 cells remained on the control level (median fluorescence 24 versus 23 units).

**Figure 3:**
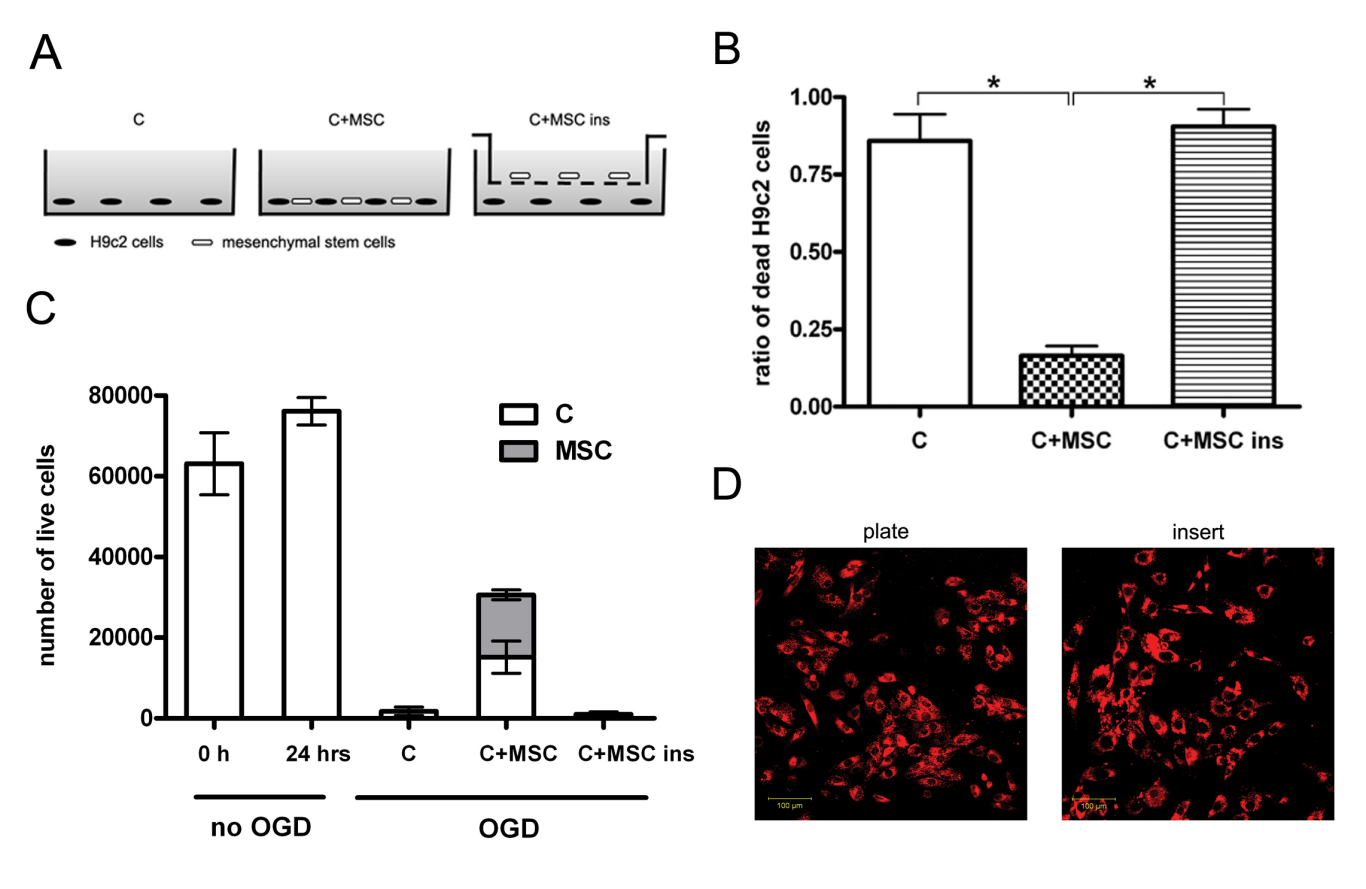
**Co-cultivation of H9c2 cells with MSCs decreased cell death**. **(A) **Experimental layouts after in vitro ischemia. **(B) **The ratio of dead H9c2 cells was significantly smaller when MSCs were added after OGD (0.85 ± 0.086 vs. 0.16 ± 0.035, n = 5), but MSCs added in cell culture inserts did not decrease significantly the ratio of dead H9c2 cells (0.90 ± 0.055, n = 5). Data represent mean ± SEM. *p < 0.05 C+MSC vs. C and C+MSC vs. C+MSC ins. (C: H9c2 cells only; C+MSC: H9c2 cells and MSCs; C+MSC ins: H9c2 cells and MSCs in cell culture inserts) **(C) **Absolute number of live H9c2 cells before and after OGD shows that before OGD the H9c2 cells were close to confluence (63,120 ± 7,694) and there was little increase in cell numbers during the next 24 hours if the cells were left to grow without OGD (76,116 ± 3,396). 24 hours after OGD the number of viable cells was very low when cultured alone or with MSCs in cell insert (1,757 ± 1,081 and 990 ± 608 respectively), which was significantly increased (15,174 ± 3,975) if MSCs added directly. **(D) **MSCs labeled with Vybrant DiD were growing on cell culture inserts in the same manner as under normal culture conditions after 24 hours of cultivation. Scale bar represents 100 μm.

Confocal microscopy showed that cardiomyoblasts cultured alone displayed the same rounded and blebbed morphology immediately following as well as 24 hours after OGD (Figure 2A). Flow cytometry analysis showed that OGD significantly increased the cell death rate in this group as shown by the enhanced ethidium homodimer fluorescence intensity (median fluorescence from 19 to 65 units, Figure 2B). Figure 2B also shows that a portion of cells remained unstained with ethidium homodimer even after 150 minutes due to the variability of the model. When MSCs were added to post-ischemic cardiomyoblasts, the morphology of the damaged cells was similar to cells cultured in normal conditions without OGD (Figure 2C). In this group, flow cytometry analysis revealed that the deleterious results of ischemia were decreased (median fluorescence 24 versus 23 units, Figure 2D). To quantify the effect of added MSCs, confocal images were used. This approach revealed that the ratio of dead H9c2 cells to all H9c2 cells in the wells 24 hours after OGD was significantly higher when the cardiomyoblasts were cultured alone compared to when healthy MSCs were added to the cultures 30 minutes after OGD (0.85 ± 0.086 vs. 0.16 ± 0.035, respectively, p < 0.05, n = 5). MSCs added in cell culture inserts, which physically separate the two cell populations growing in the same medium, failed to decrease the ratio of dead cells (0.90 ± 0.055, n = 5), indicating that the rescue factor is less likely to be a soluble one and direct cell-to-cell contact may be required (Figure 3A and 3B). The absolute number of live H9c2 cells before and after OGD and the number of added MSCs after OGD was also investigated. Before OGD H9c2 cells were close to confluence (63,120 ± 7,694) and there was little increase in cell numbers during the next 24 hours if the cells were left to grow without OGD (76,116 ± 3,396). The number of viable cells 24 hours after OGD was very low when cultured alone or with MSCs in cell insert (1,757 ± 1,081 and 990 ± 608 respectively), but significantly increased (15,174 ± 3,975) if MSCs were added directly. It can be assumed that the injured H9c2 cells were washed out during medium change so only a part of H9c2 cells remained in the wells (Figure 3C). We also examined the inserts with confocal microscopy to eliminate the possibility of decreased MSC viability on the cell culture inserts. MSCs were labeled with Vybrant DiD before seeding on the cell inserts. We found that Vybrant DiD labeled MSCs were attached to the surface of the inserts and showed normal cell morphology (Figure 3D). We also investigated whether any of the MSCs had contaminated the underlying H9c2 cardiomyoblast culture, but found no trace of MSCs among the H9c2 cells; therefore no direct cell-to-cell contact could be formed (data not shown).

### Confocal microscopy and flow cytometry

**Figure 4:**
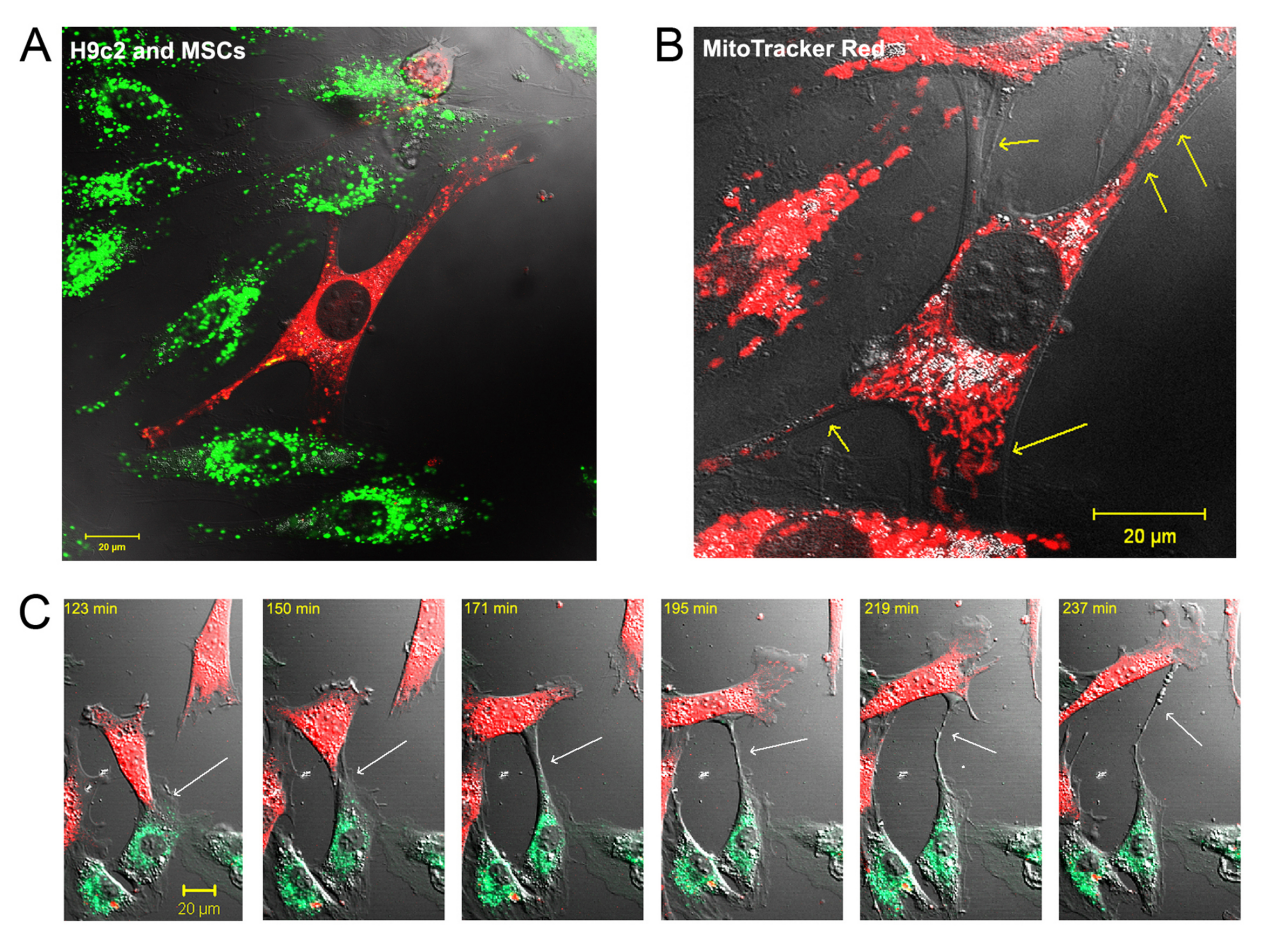
**Formation of intercellular connections after OGD**. **(A) **Nanotubular network formation was observed among DiO-labeled cardiomyoblasts (green) and DiD-labeled MSCs (red) after 24 hours of co-culture. **(B) **MitoTracker staining (red) revealed active mitochondria in the nanotubular network (yellow arrows). **(C) **Time lapse pictures of the formation of a nanotube between a DiO-labeled cardiomyoblast (green) and DiD-labeled stem cell (red).

Development of intercellular connections, so-called nanotubes, between cardiomyoblasts and MSCs during the 24 hr period after OGD was frequently observed (Figure 4A). These nanotubes were long enough to span distances of several cell diameters, and their diameters were between 200 and 500 nm. MitoTracker Red staining revealed that these nanotubes connecting stem cells to cardiomyoblasts contained functionally active mitochondria (Figure 4B). Time lapse video microscopy did not reveal a specific direction for the movement of these mitochondria in the intercellular connections and the typical time frame for the formation of a nanotube was approximately 2 hours (Figure 4C and additional file 2: video2.mov).

**Figure 5:**
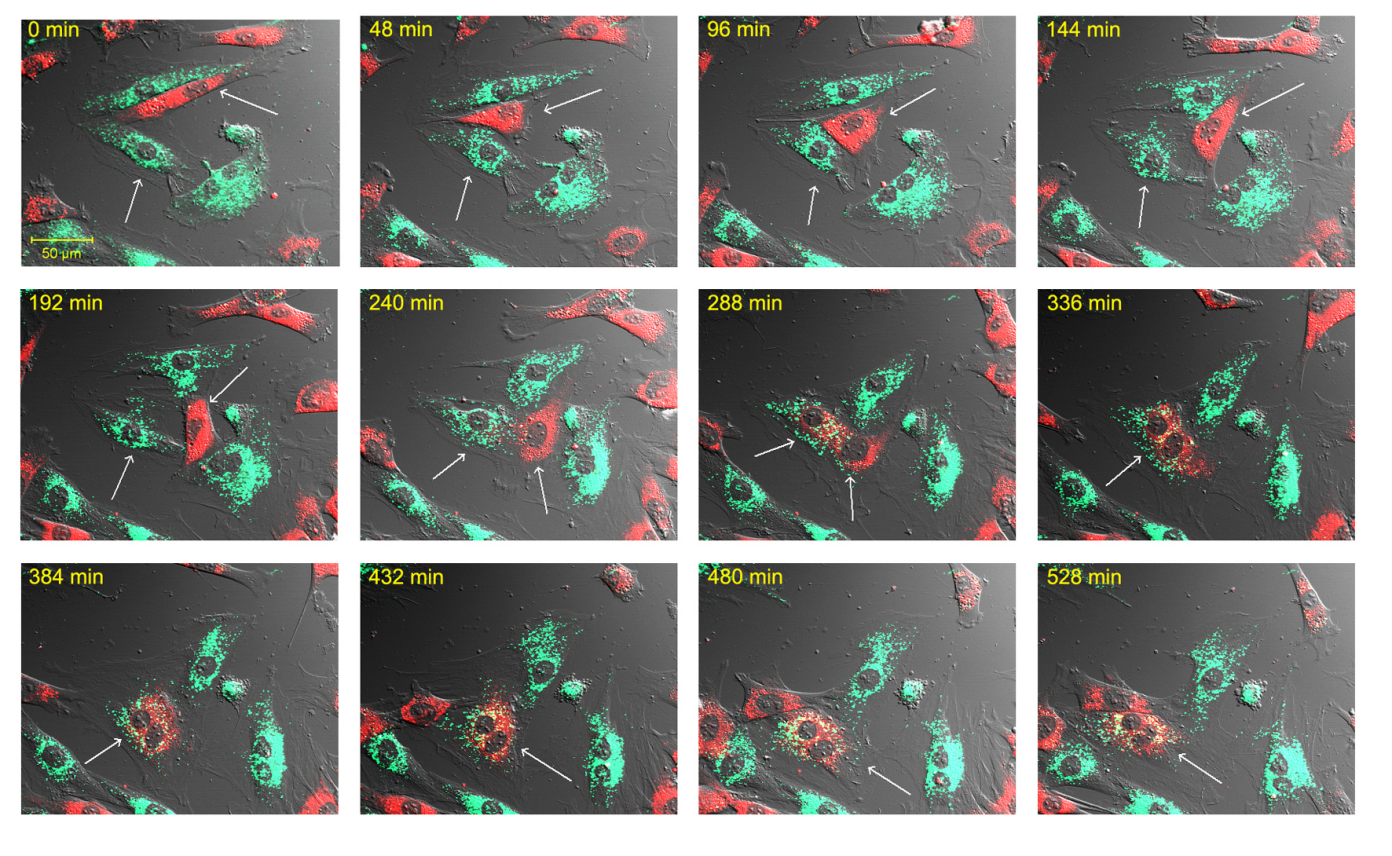
**Cell fusion of a H9c2 cell and a mesenchymal stem cell**. The time lapse pictures demonstrate steps of the fusion of a H9c2 cell (green) and a mesenchymal stem cell (red). Fused cells with double nuclei exhibit a combined yellow staining.

**Figure 6:**
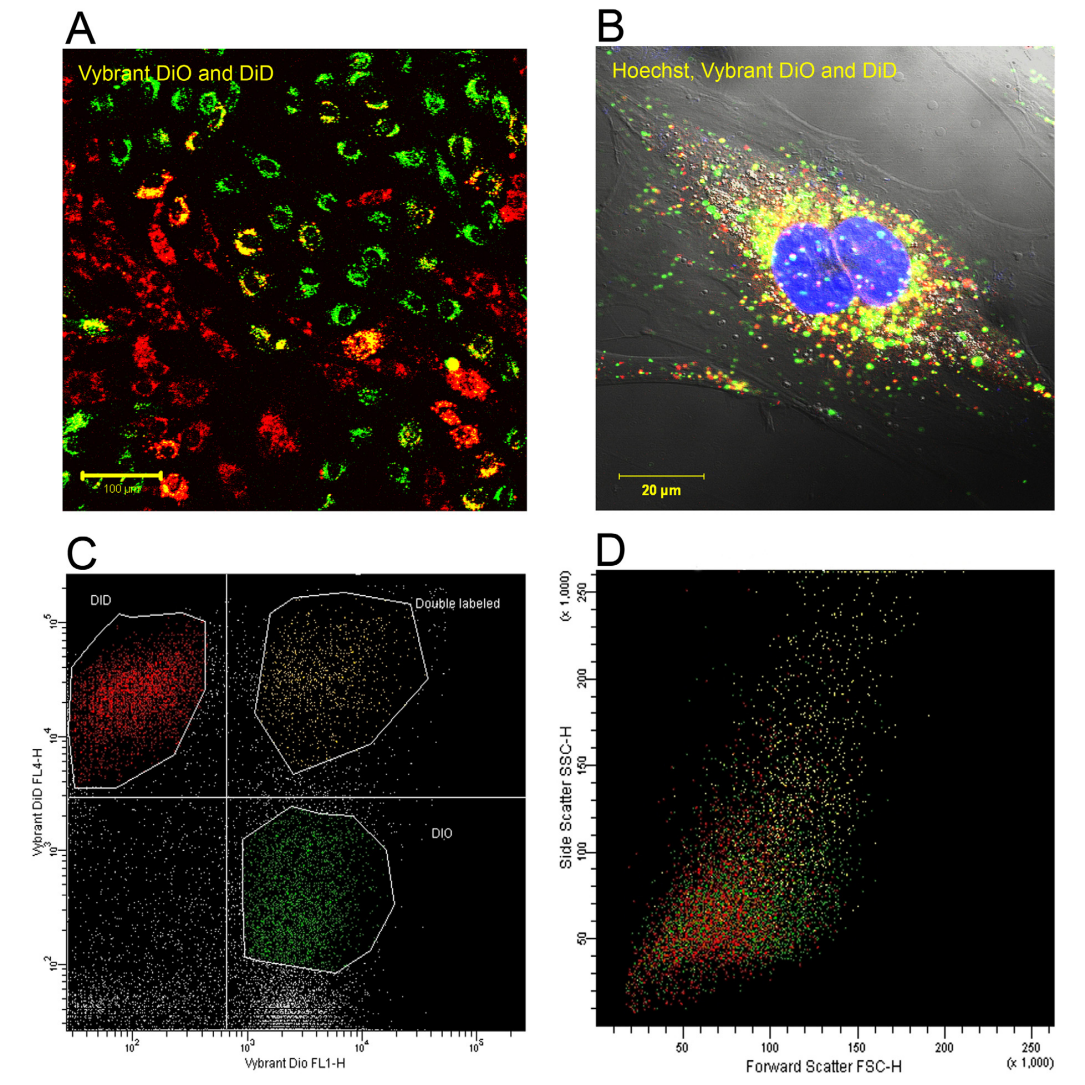
**Co-culture of H9c2 and stem cells in normal conditions**. **(A) **Cardiomyoblasts (green) and MSCs (red) after one day co-cultivation. **(B) **A representative double labeled cell with double nuclei (nuclei were stained with Hoechst). **(C) **DiO-labeled cardiomyoblasts (green) and DiD-labeled MSCs (red) analyzed with flow cytometry after one day co-cultivation. We found three different cell populations: 59.42% of DiO-labeled H9c2 cells, 30.1% of DiD-labeled MSCs and 8.14% of double labeled cells. **(D) **The distribution according to the forward scatter plot demonstrates that several double labeled yellow cells are mostly the same size as the green H9c2 cells or the red MSCs disapproving complete cell fusion.

Addition to the formation of intercellular communications, double labeled and double nuclei cells indicated that cell fusion events were present in the co-cultures. The typical time frame for a cell fusion was approximately 4 hours (Figure 5 and additional file 3: video3.mov). To examine whether cell fusion occurs in normal, non-ischemic conditions among H9c2 cells and MSCs we co-cultured these cells without OGD and found that such phenomenon also occurs among healthy cells. Flow cytometry analysis showed beside 59.42% of DiO-labeled H9c2 cells and 30.1% of DiD-labeled MSCs also 8.14% of double labeled cells. However, according to the forward and side scatter plot the distribution demonstrates that most of the double labeled cells are the same size as H9c2 cells or MSCs, suggesting that these cells are picking up the other marker through direct cell-to-cell contact (Figure 6C and 6D).

We also considered the possibility that MSCs may incorporate cell debris and thus acquire double labeling of fluorescent dyes. During the observations performed with time-lapse video microscopy we frequently saw that healthy cells contacted and moved around cell debris and apoptotic bodies in the culture dish, however, phagocytosis was not observed. The cells which contacted differentially stained cell debris did not pick up any fluorescent signal from the other, indicating that the double labeling of cells arose from a specific and controlled mechanism rather than cross-contamination. This is further strengthened by the observation that not all cell-to-cell connection resulted in dye transfer. (additional file 4: video4.mov)

## Discussion

We report that healthy mesenchymal stem cells are capable of rescuing post-ischemic cardiomyoblasts from cell death through a mechanism not yet implicated in the effects of stem cells after ischemic conditions. Thus, the beneficial effect of stem cell grafting may be based not only on improved neovascularisation and replacement of lost cells but on rescuing the damaged cells of the host as well.

The most likely explanation of the beneficial effects of MSC co-culture is that these cells improve the chances of the damaged H9c2 cells to restore their function and prevent later cell death. Ethidium homodimer has been shown to stain not only dead but damaged cells as well [23]. Thus, although most H9c2 cells were stained by ethidium homodimer after OGD (Figure 1B), many cells were probably only reversibly damaged. An alternative explanation of our results could be an increased replication of surviving H9c2 cells. However, the nearly ten-fold difference in the number of viable H9c2 cells between our experimental groups 24 hr after OGD (Figure 3C) and the normal doubling time of these cells make this possibility unlikely to explain the difference.

The used in vitro ischemia model demonstrates that the beneficial effects of MSC co-culture seem to be dependent on direct cell-to-cell connections and intercellular nanotubes.

Nanotube formation has already been shown to occur among endothelial progenitor cells, cardiomyocytes [20,24], immune cells and other lineages [25,26]. The characterization of nanotubes revealed that these filaments contain actin and in some cases, microsomes or mitochondria [27]. We found that this phenomenon occurs frequently between cardiomyoblasts and mesenchymal stem cells. Stem cells failed to rescue post-ischemic cardiomyoblasts when intercellular connections were blocked by a physical barrier. These observations indicate that intercellular connections work toward the survival of cells both during and after ischemia, however, the underlying mechanisms may be slightly different.

One plausible mechanism for the rescuing effect is that transplanted cells improve regeneration through secreting paracrine factors [14,28-31]. However, results from our experiments with the plate insert show that paracrine factors secreted by the cells are probably too low in our system to have any beneficial effect on these severely damaged cells. This in vitro experimental setup allows the investigation of cell-to-cell contacts, however, it cannot rule out that paracrine effects play a significant role in a more physiological in vivo setting. The time frame of the experimental protocol is also important. In our experiments we added the cells at an early time point and terminated the experiment before significant differentiation can occur. During a later time point the effect of paracrine factors is probably much more important especially in the differentiation process as shown by several other authors [6,11].

Cell fusion is another phenomenon which is frequently observed in co-culture studies and in some cases, in in vivo experiments as well [15,32,33]. Several studies have shown that cell fusion can result in transdifferentiation, thus offering an alternative mechanism by which grafted cells improve the infarcted myocardium. Using videomicroscopy we also found several double labeled, double nuclei cells indicative of cell fusion. However, cell fusion showed high variations among different culture and detection techniques, and therefore extensive cell fusion as an in vitro artefact cannot be ruled out [14,34,35]. During our investigations we only observed a few unquestionable cell fusions which cannot account for the rescue of the high number of damaged cardiomyoblasts [36].

We also found double labeled cells without double nuclei in the co-culture of cardiomyoblasts and stem cells after 24 hours. The double labeling of these cells may be the result of direct cell-to cell connections. During these periods of connection, cells are able to exchange membrane parts and Vybrant dye molecules can drift from one cell to another. Movement of dye molecules from one cell to another through gap junction connections is precluded because the lipophilic Vybrant dyes are high molecular weight stains and cannot permeate through gap junctions [34]. Driesen et al. [19] showed that low molecular weight tracers such as calcein-AM get from one cell to another through gap junctions, and high molecular weight tracers by partial cell fusion, thus the conclusion may be drawn that dye transfers after 24 hours in our experiment are most probably the results of direct cell-to-cell connections. Still, gap junctions may create an opportunity for grafted cells to interact with the host tissues [37,38]. In the present experimental model most of cell-to-cell interactions were short-lived tubular connections, which formed a constantly changing web between the two investigated cell types.

Our experimental model was devised to investigate acute effects with high temporal and spatial resolution, therefore ruling out differentiation, which occurs over time. Moreover, an in vitro transplantation model in a cell culture system cannot mimic the 3-dimensional tissue where cell-to-cell connections are different. These circumstances obviously limit the conclusions drawn from our results. On the other hand, this experimental setting was necessary and favorable to investigate short-term cellular interactions.

## Conclusions

The present study highlights that stem cell grafting may be beneficial through an acute, direct mechanism which saves damaged cardiomyoblasts. Novel grafting protocols can harness this effect, which raises the possibility that stem cells given early and locally can preserve heart tissue rather than simply help to replace what is already lost.

## Authors' contributions

ACS and ZSL conceived the study. ACS completed the majority of the confocal microscopy experiments, made the flow cytometry measurements, performed statistical analysis, and helped draft the manuscript. EP helped draft the manuscript and performed statistical analysis. EMH participated in the flow cytometer measurements. KL participated in the time lapse video microscopy experiments and helped draft the manuscript. In addition to collaborating on the conception of the study, ZSL participated in the study design, provided coordination among the researchers and experiments, and helped draft the manuscript. All authors read and approved the final manuscript.

## Supplementary Material

Additional file 1 Follow up of 150 minutes long OGD on cardiomyoblasts with time lapse video microscopy. Click here for file

Additional file 2 Nanotubular network formation among DiO-labeled cardiomyoblasts (green) and DiD-labeled MSCs (red) after 24 hours of co-culture. Click here for file

Additional file 3 Time lapse video microscopy pictures demonstrates fusion of a H9c2 cell (green) and a mesenchymal stem cell (red). Click here for file

Additional file 4 14 hours of time lapse video microscopy of H9c2 cells (green) and MSCs (red) after OGD. Click here for file
